# The Effects of Urban Green Space on Depressive Symptoms of Mid-Aged and Elderly Urban Residents in China: Evidence from the China Health and Retirement Longitudinal Study

**DOI:** 10.3390/ijerph19020717

**Published:** 2022-01-10

**Authors:** Rui Zhou, Ying-Jing Zheng, Jing-Yi Yun, Hong-Mei Wang

**Affiliations:** Department of Social Medicine of School of Public Health, and Department of Pharmacy of the First Affiliated Hospital, School of Medicine, Zhejiang University, Hangzhou 310058, China; zhou_rui@zju.edu.cn (R.Z.); zhengyingjing@zju.edu.cn (Y.-J.Z.); xyun@zju.edu.cn (J.-Y.Y.)

**Keywords:** depression, green space, mid-aged and elderly, China

## Abstract

The aim of this study is to assess the impacts of urban green space on depressive symptoms among Chinese urban residents aged 45 and older. In total, 7397 urban respondents were included in this study. Each respondent participated in the China Health and Retirement Longitudinal Study Wave 3 (2015). Environmental-level variables were retrieved from the National Bureau of Statistics database. Both unadjusted and adjusted methods were used in the multilevel regression analysis. Almost one-third of the sample population suffered from depressive symptoms (31.20%). The multilevel logistic regression model showed that green coverage ratio of city-built districts is negatively associated with the prevalence of depressive symptoms among urban mid-aged (OR = 0.79, *p* < 0.05) and elderly (OR = 0.75, *p* < 0.05) residents, and the public recreational green space helps to reduce elderly people’s depressive symptoms (OR = 0.77, *p* < 0.05). This study adds insights about the impact of green space and other environmental factors on depressive symptoms among mid-aged and elderly urban dwellers. It is important to provide enough and accessible overall urban green spaces; additionally, attention should also be paid to specific green space forms such as public recreational green space.

## 1. Introduction

Depression, one of the most common mental disorders in many countries, affects approximately 7% of the elderly population worldwide [1]. The situation among the mid-aged population is also not optimistic; prevalence of depression among females and males aged 45–59 is more than 7% and 5%, respectively [2]. Depression contributes greatly to daily function impairment and increases the suicide rate [3,4]. There is some evidence to suggest that prevalence of depressive disorders among urban residents in high-income countries is higher than their rural counterparts [5,6]. Although the situation in China has been reported to be exactly the opposite [7,8], the rate of depressive symptoms among Chinese urban populations is dramatically higher than in other low- and middle-income countries and Asian countries [9,10,11]. China has about 60% of its population living in urban areas in 2018 [12], and the percentage of the elderly population living in cities rose from 26.3% in 2006 to 52.0% in 2015 [13,14]. Therefore, the depressive problems of urban residents also need particular attention in China.

Existing studies have found a high rate of depressive symptoms among mid-aged and elderly population in China and explored the association between depression and individual-level factors such as gender, income, health status, and financial transfers [7,8,15,16], while it is increasingly important to explore environmental risk factors for this major mental health issue against the backdrop of urbanization [17].

Previous studies initially focused on urban environment and mental health have reported that denser urban population [18] and exposure to noise pollution [19] and air pollution [20] were related to increased depressive symptoms. Furthermore, exposure to natural environment including parks and other green spaces seems to play a positive role in relieving depression of urban residents [11,21]. Natural green resources can help improve mental health by encouraging physical activity [22] and social interaction [23]. The relations between green space and mental health can vary with availability, typology, and several other aspects of green space [24,25]. Specific types of green space may be more strongly associated with health outcomes than overall quantity of green space [26], and health effects could differ by type of green space; for example, trees were found to have superior benefits than grassland in improving mental health [27]. However, previous studies in this field usually concern people of all ages [28,29], youth [30,31,32,33], university students [34,35,36], or patients with specific diseases [37,38,39], while only limited researches focus on mid-aged and elderly urban residents [11,40]. Moreover, most studies have been conducted in several developed countries, while little attention has been paid to the context of developing countries with undergoing rapid urbanization such as China.

This study will investigate the association between urban green space and depressive symptoms among urban mid-aged and elderly residents in China, hoping to provide evidence for the formulation of effective policies to cope with urbanization and population aging. The mid-aged and the elderly are both at high risk of developing depressive symptoms. Meanwhile, there could be substantial differences in activity patterns and interaction with the environment in adults at different life stages [41,42,43], the impacts of green space may also vary across age groups. Therefore, our study included both the mid-aged and the elderly respondents and described the impacts of green space on respondents of different age ranges, respectively. For older citizens, prior studies emphasized park as a special type of urban green space [40], and immediate surroundings with greenery are vital for the elderly if they experience physical health problems [44,45]. We therefore examined per capita public recreational green space (including park) and the proportion of green coverage area in the total area of city-built districts in this study.

## 2. Materials and Methods

### 2.1. Study Design

This study was a secondary analysis of data collected from the China Health and Retirement Longitudinal Study (CHARLS) Wave 3 (2015). The data and relevant files can be found in the China Health and Retirement Longitudinal Study official website repository, http://charls.pku.edu.cn/index/zh-cn.html (accessed on 10 December 2019). The CHARLS is a cross-national, population-based research project exploring extensive topics related to health and socioeconomic status among people aged 45 and older in China. A multi-stage stratified probability proportional to size (PPS) sampling design was employed to select respondents from 450 villages in 150 county-level units of 28 provinces in China. The baseline survey was launched in 2011–2012 and follow-up surveys were conducted every two or three years. The CHARLS was conducted according to the guidelines of the Declaration of Helsinki and approved by the Institutional Review Board at Peking University (IRB00001052-11015). Further detailed descriptions of CHARLS have been published elsewhere [20,46,47]. The variable community ID recorded respondents’ household living regions at the community level, based on which the CHARLS classified respondents’ places of residence into urban and rural according to National Bureau of Statistics of the People’s Republic of China. We restricted the sample to people aged 45 and older living in urban areas and having full information on the included variables, resulting in a sample size of 7397 respondents from 213 neighborhoods nested within 103 cities, among which there were 3814 mid-aged people aged between 45 and 59 and 3583 elderly people aged 60 and older.

Depressive symptoms were measured by the 10-item short form of the Center for Epidemiological Studies-Depression Scale (CESD-10) in CHARLS. All items are rated on a four-point Likert scale ranging from 0 (rarely or none of the time) to 3 (most or all of the time). The total score values range from 0 to 30, with higher scores indicating greater depressive symptoms, and a score of 10 or higher was used as a cut-off point for depressive symptoms [48]. CESD-10 has been proved to have adequate psychometric properties elsewhere [49]. A Cronbach’s alpha coefficient value of 0.71 was found for the scale in this study.

### 2.2. Measures

#### 2.2.1. Explanatory Variables

Urban green space is defined as including parks, affiliated green space, production green space, protective green space, etc. [36,50] To assess the amount of green space, we selected two existing city-level variables: one is the per capita public recreational green space (m^2^/person) for entertainment, sports, and social interaction, and the other one is the proportion of green coverage area that represents the overall green coverage in the total area of city built districts (hectares/Km^2^). These data were derived from the 2015 China Urban Construction Statistical Yearbook [50]. Following the approach described in other similar studies [51,52,53], the two explanatory variables were divided into three levels using tertiles (Table 1). Details of the three levels are as follows: (1) per capita public recreational green space—low level: 6.98–14.90 m^2^/person; medium level: 15.07–19.85 m^2^/person; high level: 21.82–38.11 m^2^/person, (2) green coverage area in built up area of city—low level: 18.14–37.67 hectare/Km^2^; medium level: 38.03–42.95 hectare/Km^2^; high level: 43.33–52.02 hectare/Km^2^.

#### 2.2.2. Control Variables

Age, gender, marital status, education, and work were included as demographic variables. Participants were divided into two age groups: 45–59 and 60 and above. Marital status was grouped as being married or cohabitated versus separated, divorced, widowed, or never married. Education was categorized as primary education or below, middle school, senior high school, and associate degree or above. Based on a series of questions about job status, the variable work was generated as a binary variable indicating whether the respondents had a job at the time of survey.

Co-morbidity condition, physical function, and participation of outdoor activity were included as health-related factors. Respondents were asked if they had been diagnosed with any of the listed 14 chronic diseases, and co-morbidity was grouped as having no disease, suffering from one disease, or having co-morbidity. Physical function was measured by a short version of Instrumental Activities of Daily Life Scale (IADL). The short form consists of six items, and all items are rated on a four-point scale ranging from 1 (having no difficulty) to 4 (cannot do it); people who scored higher than 6 had a different degree of physical function damage [54]. Internal consistency for this short form measured by Cronbach’s α was 0.84. Participation of outdoor activity was grouped as yes or no to the question “Have you participated in activities such as dancing, exercising, etc., in the last month?” [55].

City type, city GDP (billion RMB), and population density (persons/Km^2^) were involved as city-level environmental control variables, which have been proved associated with urban green space [56,57,58]. In this study, the city was categorized by administrative hierarchy into four types: municipality directly under the central government, vice-provincial city, prefecture-level city, and county-level city. Development of a city can be closely associated with its administrative level. Cities with higher administrative level tend to be larger, more prosperous, and at advantage of resource allocation [59]. The city GDP (billion RMB) and population density (persons/Km^2^) can be obtained from the 2016 China City Statistical Yearbook and the 2015 China Urban Construction Statistical Yearbook.

### 2.3. Statistical Analysis

In this study, people aged between 45–59 and those aged 60 and older were analyzed, respectively. Descriptive analysis was used to give a general overview of respondents’ characteristics; the results were presented in the form of percentage for categorical variables and means and standard deviation (SD) for continuous variables. Logistic analysis results were given to identify the associations between depressive symptoms and each of the individual level variables (including demographic and health-related variables) and environmental control variables. Odds ratios (ORs) and the corresponding 95% confidence intervals (CIs) were reported. Relations between depression and respondents’ characteristics are examined to reflect the representativeness of our sample.

The multilevel logistic regression models to predict depressive symptom were then calculated with individuals nested within cities. The unadjusted model including only the green space indicators was first built, based on which further models controlling for individual level and environmental control variables were constructed to investigate the influence of green space more specifically. The Akaike Information Criterion (AIC) is an estimate of model goodness-of-fit, according to which models that can fit the data with the fewest free parameters are supposed to be the best. The lower the AIC of a model, the better the goodness-of-fit [60]. Descriptive analysis and binary logistic regression analysis were performed in SPSS version 24 (IBM Corp., 2016, Armonk, NY, USA) and the multilevel logistic regression models were performed by Stata version 15 (Stata Corp., 2017, College Station, TX, USA).

## 3. Results

### 3.1. Study Sample Characteristics

A total of 7397 people was included, among which 3814 (51.56%) people were mid-aged and 3583 (48.44%) people were elderly. Of these respondents, the mean age was 59.93 (SD = 10.16), and the average CESD-10 score was 7.86 (SD = 5.11). Table 2 demonstrates the characteristics of the study sample. One-half of the sample were female (51.29%), and more than 80% were married or cohabitated (86.97%). Nearly half of the respondents (48.65%) had primary school or below education, and less than a third of the respondents (22.48%) had a high school degree or a higher qualification. In total, 2116 (28.61%) respondents had two or more chronic diseases, and 21.32% of the respondents had physical function damage. Over four-fifths of the respondents (87.67%) did not engage in outdoor activity in the last month. An overview of characteristics of the cities is provided in Table 2 as well. The majority of respondents lived in prefecture-level cities (80.52%). Nearly three-quarters of the respondents (74.64%) lived in economically less developed cities, and 5118 (69.19%) respondents lived in cities with relatively sparse population.

### 3.2. Binary Analysis of Respondents’ Characteristics with Depressive Symptoms

Almost one-third of all respondents suffered from depressive symptoms (31.20%). Table 3 illustrates the binary analysis of individual level factors and environmental control factors with depressive symptoms.

For the mid-aged group, the correlates for having a high risk of depressive symptoms were: being female, having had a lower level of education, not married/cohabitated, having chronic diseases, physical function damage, and living in a city with a less developed economy. The correlates for having depressive symptoms in the elderly group slightly differed from those in the mid-aged group. Elderly respondents who have a job, living in a city of a lower administrative level reported higher risk of depression, while marital status did not have a significant effect on depressive symptoms.

### 3.3. Association between Urban Green Space Indicators with Depressive Symptoms

Table 4 shows the ORs (95% CI) of depressive symptoms among the sample population according to the per capita public recreational green space and green coverage area in built up area of city.

In the unadjusted model, only medium level of green coverage rate was associated with reduced depressive symptoms (OR = 0.74; CI: 0.58, 0.95) in the mid-aged group. When green coverage rate increased to medium level, the risk of depression in elderly people was significantly reduced by 33% (OR = 0.67; CI: 0.53, 0.86). Compared with medium level, high level of green coverage rate did not further significantly reduce depressive symptoms (OR = 0.67; CI: 0.49, 0.92). Per capita public recreational green space was not associated with depressive symptoms in the two age groups.

In Models 1, 2, and 3, potential variables were adjusted relative to the unadjusted model. Adding variables leads to a decrease in AIC scores and thus an improvement in model goodness-of-fit. After adjustment by demographic and health-related variables (Model 2), the per capita public recreational green space was associated with less depressive symptoms of the elderly, with depression reduced by 23% (OR = 0.77; CI: 0.59, 0.99). However, the effects of coverage rate on depressive symptoms of the mid-aged and elderly both became smaller.

Model 3 (a), a full model controlling for both individual level and environmental control variables to reduce the impact of selection bias, had the lowest AIC and thus the most appropriate model fit.

The positive effects of green space were less salient in mid-aged group. The medium level of the green coverage rate was associated with less depression in the mid-aged group (OR = 0.79; CI: 0.62, 0.99). Per capita public recreational green space, however, was not statistically significant at any level. The risk of depression among the elderly was reduced by 23% when the per capita public recreational green space reached a medium level (OR = 0.77; CI: 0.61, 0.99). Model 3 (a) also indicated that medium level of green coverage rate was significantly associated with a reduction in depression of elderly residents, with depression reduced by 25% (OR = 0.75; CI: 0.60, 0.94). A similar result occurred when green coverage rate increased to high level. An interaction term between per capita public recreational green space and green coverage area in built up areas of cities was examined in model 3 (b), which was not significantly associated with depression.

## 4. Discussion

As a result of social and economic development, more than half of the Chinese population now live in urban areas, and the proportion of elderly residents has increased greatly [12,13,14]. Given that elderly urban residents are at high risk of developing depressive symptoms, more attention should be paid to the mental health status of the ever-increasing aging population in urban areas. Based on a large-scale, nationwide representative survey, this study described depressive symptoms among mid-aged and elderly urban residents in China and examined the associated factors. A high prevalence of depressive symptoms was found among Chinese urban residents aged 45 and over, which is consistent with the recent literature on China [7,8] but higher than that in other Asian countries, such as south Korea [11] and Indonesia [9]. The factors relevant to depression differed between the mid-aged and elderly groups in this study.

We found the prevalence of depressive symptoms was higher among women and respondents who lived in a city with a less developed economy. Similar to previous studies, lower education level, having chronic diseases, and physical function damage contributed greatly to depressive symptoms among the mid-aged and the elderly [7,11]. However, this study did not find a significant association between outdoor activity and depressive symptoms, when respondents only reported whether they participated in activities such as dancing and exercising in the last month. Furthermore, marital status was not found to have an influence on depression for the elderly, and employment status as well as the administrative hierarchy of city were not predictors of depressive symptoms for mid-aged people. The exclusion of participants with missing information may help explain why these factors cannot exert influence on the mid-aged and the elderly groups simultaneously.

In recent years, city design has started to focus on promoting elderly-friendly environments where the elderly can have more opportunities to make contact with nature such as green spaces [61,62,63]. Great emphasis has been made on urban green spaces, which have potential benefits for physical health outcomes, such as weight status and chronic morbidities [64,65]; evidence also indicated a negative association between green space and the impacts of COVID-19 [66,67]. Additionally, green spaces have been consistently found to play an active role in promoting mental health [11,68,69]. This study examined whether total green space or specific types of green space were associated with fewer depressive symptoms among mid-aged and elderly urban residents after controlling for individual level factors and environmental control variables.

The green coverage ratio of city-built districts was associated with fewer depressive symptoms among mid-aged and elderly urban residents, which is in accordance with previous studies reporting that the higher the rate of greenery in a city, the less stress and fewer symptoms of depression among its elderly residents [11,70,71]. Increase in urban green spaces is thus encouraged as the urban population is aging. It should be noted that green coverage ratio of city-built districts showed more positive effects in reducing depression in the elderly. Contributors of depression and the corresponding significant influencing factors could differ at different life stages [72]. The influence of environmental factors for the mid-aged may be undermined by other more significant aspects such as socioeconomic status, as they shoulder a heavy burden of work and family support [73,74]. It is interesting that the per capita public recreational green space including park only helped to relieve depressive symptoms for the elderly. A qualitative study showed that larger green spaces were perceived to be better and more attractive than smaller green space, and senior citizens were more likely to use larger green spaces such as parks [40], while the majority of the mid-aged group were still at work and had fewer opportunities to use public recreational green space. Considering that the increase in the total green space can reduce depressive symptoms, further research should investigate the relationship between green spaces in residential and work areas, and depressive symptoms for the mid-aged. Additionally, the government should also consider establishing appropriate social welfare policies for the elderly to promote them to interact more with public recreational green spaces.

### Strengths and Limitations

This study does have some important strengths. First, owing to a series of strict quality control measures (e.g., global positioning system matching) of CHARLS, the sample in this study was representative of urban mid-aged and elderly residents. Furthermore, different from other studies that focus on high-income countries [75] or megacities such as Beijing [76,77], most respondents in this study lived in prefecture-level cities in China. The results can thus help us understand the situation of middle-sized cities and provide practical suggestions for welfare policies in relation to urban green space. Secondly, multilevel analyses were used to account for the relationship between depressive symptoms and provision of green space, which allowed us to identify age-specific patterns of association between green space and depressive symptoms. Moreover, our study makes a supplement to existing studies in this field by first time exploring the impacts of public recreational green space as a specific form of urban green space on depressive symptoms in Chinese cities.

There are also several limitations with this study. First, our study was cross-sectional, which prevents us from drawing causal associations between green space and depression. Second, due to the absence of information on respondents’ addresses, respondents’ exposure to green space could not be measured precisely in terms of individual residential area. However, official statistics of green space at the city level could be accepted given the common tendency for people to interact with a range of different places in their city. Third, this study only focused on the amount of green space but did not investigate the quality of green space, which could also play an important role in improving mental health [78,79]. Measurement of quality of green space should be included in the future to describe the effects of green space on depression more comprehensively. Fourth, the variable participation of outdoor activity is based on a single question in the CHARLS questionnaire, indicating whether respondents participate in specific activities or not; further research should include variables measuring amount and type of outdoor activities to assess participation of outdoor activity more accurately and comprehensively.

## 5. Conclusions

This study adds insights about the impact of green space and other environmental factors on depressive symptoms among mid-aged and elderly urban dwellers. High green coverage ratio of city-built districts might protect both mid-aged and elderly urban residents against depressive symptoms. Meanwhile, as a specific quantitative indicator, the per capita public recreational green space including park was associated with fewer depressive symptoms among the elderly. These findings may provide empirical evidence for mental health policy-making. Enough and accessible overall urban green space should be provided, and specific type of green spaces such as public recreational green space deserve particular attention; additionally, appropriate social welfare policies and nature-based activities can be developed to promote interaction with existing green resources of urban residents. To deepen the understanding of impacts of green space on mental health and improve green space planning and design, further research exploring impacts of different typologies of green space and the pathways that link green space and mental health is recommended.

## Figures and Tables

**Table 1:** Descriptive statistics of urban green space indicators.

Category	Per Capita Public Recreational Green Space (m^2^/person)			Green Coverage Area in Built Up Area of City (hectare/Km^2^)		
	Mean (SD)	Min	Max	Mean (SD)	Min	Max
Low Level	11.45 (±2.12)	6.98	14.90	33.35 (±4.48)	18.14	37.67
Medium Level	17.27 (±1.48)	15.07	19.85	40.12 (±1.18)	38.03	42.95
High Level	25.53 (±5.50)	21.82	38.11	45.82 (±2.55)	43.33	52.02
Total	13.52 (±4.50)	6.98	38.11	39.78 (±4.76)	18.14	52.02

**Table 2:** Study sample characteristics.

Variables	Category	*N*	%
Age	45–60	3814	51.56
	≥60	3583	48.44
Gender	Male	3603	48.71
	Female	3794	51.29
Marital Status	Married and Cohabitated	6433	86.97
	Otherwise	964	13.03
Education level	Primary education or below	3599	48.65
	Middle school	2135	28.86
	Senior high school	1197	16.18
	Associate degree or above	466	6.30
Work	Yes	5630	76.11
	No	1767	23.89
Co-morbidity	No disease	3528	47.70
	Only one disease	1753	23.70
	≥two diseases	2116	28.61
Physical function damage	Yes	1577	21.32
	No	5820	78.68
Participation of outdoor activity	Yes	912	12.33
	No	6485	87.67
City Type	Municipality Directly under the Central Government	280	3.79
	Vice-provincial City	769	10.40
	Prefecture-level City	5956	80.52
	County-level City	392	5.30
Community city GDP (billion RMB)	<500	5521	74.64
	500–1000	1146	15.49
	≥1000	730	9.87
Population density (persons/Km^2^)	<2500	5118	69.19
	2500–5000	1985	26.84
	≥5000	294	3.97

**Table 3:** ORs (95% CI) of depressive symptoms in relation to individual and environmental factors by age group (*N* = 7396).

Variables	Category	Unadjusted OR (95% CI)	
		Mid-Age Group	Elderly Group
Gender	Male	1.00	1.00
	Female	2.024 (1.758, 2.330) ***	1.744 (1.511, 2.013) ***
Marital status	Married and Cohabitated	1.00	1.00
	Separated, Divorced, Widowed, and Never married	1.496 (1.144, 1.956) **	1.144 (0.962, 1.360)
Education level	Primary education or below	1.00	1.00
	Middle school	0.790 (0.673, 0.927) *	0.826 (0.690, 0.989) *
	Senior high school	0.666 (0.550, 0.807) ***	0.627 (0.489, 0.804) ***
	Associate degree or above	0.422 (0.310, 0.575) ***	0.631 (0.437, 0.910) *
Work	Yes	1.00	1.00
	No	0.916 (0.734, 1.143)	0.671 (0.578, 0.780) ***
Co-morbidity	No disease	1.00	1.00
	Only one disease	1.354 (1.142, 1.607) ***	1.281 (1.060, 1.548) *
	≥two diseases	1.624 (1.363, 1.935) ***	1.883 (1.595, 2.223) ***
Physical function damage	Yes	1.00	1.00
	No	0.354 (0.288, 0.434) ***	0.478 (0.412, 0.554) ***
Participation of outdoor activity	Yes	1.00	1.00
	No	0.936 (0.765, 1.146)	1.045 (0.838, 1.304)
City type	Municipality Directly under the Central Government	1.00	1.00
	Vice-provincial City	0.705 (0.460, 1.082)	1.253 (0.794, 1.976)
	Prefecture-level City	0.952 (0.657, 1.380)	1.732 (1.161, 2.853) **
	County-level City	1.342 (0.853, 2.112)	1.660 (1.001, 2.754) *
Community city GDP (Billion RMB)	<500	1.00	1.00
	500-1000	0.636 (0.516, 0.784) ***	0.514 (0.414, 0.638) ***
	≥1000	0.916 (0.724, 1.159)	0.749 (0.588, 0.955) *
Population density (persons/ Km^2^)	<2500	1.00	1.00
	2500-5000	1.086 (0.921, 1.086)	1.006 (0.848, 1.195)
	≥5000	0.903 (0.763, 1.068)	1.089 (0.918, 1.290)

* *p* < 0.05; ** *p* <0.01; *** *p* < 0.001.

**Table 4:** Results of multilevel logistic models of depressive symptoms by age group.

Variables	Category	Unadjusted Model	Adjusted Model			
			Model 1	Model 2	Mode 3 (a)	Model 3 (b)
		Mid-Aged Group				
Per capita public recreational green space (m^2^/person)	Low Level	Reference	Reference	Reference	Reference	Reference
	Medium Level	0.99 (0.76, 1.29)	1.01(0.78, 1.31)	1.04 (0.81, 1.34)	0.99 (0.77, 1.27)	1.17 (0.84, 1.61)
	High Level	1.08 (0.72, 1.63)	1.07(0.72, 1.59)	1.05 (0.72, 1.54)	1.07 (0.73, 1.55)	1.39 (0.84, 2.29)
Green coverage area in built up area of city (hectare/Km^2^)	Low Level	Reference	Reference	Reference	Reference	Reference
	Medium Level	0.74 (0.58, 0.95) *	0.73 (0.57, 0.93) *	0.73 (0.58, 0.92) **	0.79 (0.62, 0.99) *	0.83 (0.65, 1.06)
	High Level	0.78 (0.57, 1.07)	0.79 (0.58, 1.08)	0.79 (0.59, 1.06)	0.86 (0.64, 1.15)	1.02 (0.71, 1.46)
AIC		4687.427	4572.72	4503.146	4500.684	4502.782
Variables	Category	Elderly group				
Per capita public recreational green space (m^2^/person)	Low Level	Reference	Reference	Reference	Reference	Reference
	Medium Level	0.78 (0.59, 1.01)	0.76 (0.59, 0.99) *	0.77 (0.59, 0.99) *	0.77 (0.61, 0.99) *	0.82 (0.58, 1.15)
	High Level	1.40 (0.92, 2.15)	1.33 (0.88, 2.03)	1.23 (0.81, 1.85)	1.16 (0.79, 1.71)	1.25 (0.74, 2.09)
Green coverage area in built up area of city (hectare/Km^2^)	Low Level	Reference	Reference	Reference	Reference	Reference
	Medium Level	0.67 (0.53, 0.86) **	0.66 (0.52, 0.84) **	0.70 (0.56, 0.89) **	0.75 (0.60, 0.94) *	0.76 (0.60, 0.96) *
	High Level	0.67 (0.49, 0.92) *	0.67 (0.49, 0.92) *	0.71 (0.52, 0.97) *	0.75 (0.57, 0.99) *	0.77 (0.55, 1.09)
AIC		4419.798	4356.739	4266.486	4255.97	4257.827

Model 1: adjusted for demographic factors (i.e., gender, marital status, education level and work); Model 2: adjusted for model 1 + health-related factors (i.e., comorbidity, physical function, and outdoor activities); Model 3 (a): adjusted for model 2 + environmental control factors (i.e., city type, city GDP, population density); Model 3 (b): adjusted for Model 3 (a) + interaction of per capita public recreational green space and green coverage area in built up area of city, both interaction variable were not significant, *p* mid-aged = 0.124 > 0.05, *p* elderly = 0.706 > 0.05; * *p* < 0.05, ** *p* < 0.01.

## Data Availability

Publicly available datasets were analyzed in this study. This data can be found here: (http://charls.pku.edu.cn/pages/data/2015-charls-wave4/en.html (accessed on 10 December 2019)).
